# Recurrence of cervical intraepithelial lesions after thermo-coagulation in HIV-positive and HIV-negative Nigerian women

**DOI:** 10.1186/s12905-016-0304-8

**Published:** 2016-05-11

**Authors:** Emmanuel A. Oga, Jessica P. Brown, Clayton Brown, Eileen Dareng, Victor Adekanmbi, Michael Odutola, Olayinka Olaniyan, Richard Offiong, Kayode Obende, Ayodele Stephen Adewole, Achara Peter, Patrick Dakum, Clement Adebamowo

**Affiliations:** Department of Epidemiology and Public Health, University of Maryland School of Medicine, Baltimore, MD 21201 USA; Institute of Human Virology Nigeria (IHVN), Abuja, Nigeria; National Hospital, Abuja, Nigeria; University of Abuja Teaching Hospital, Gwagwalada, Nigeria; Garki Hospital, Abuja, Nigeria; Mother and Child Hospital, Ondo, Nigeria; Federal Medical Centre, Keffi, Nigeria; University of Maryland Marlene and Stewart Greenebaum Cancer Center, Baltimore, MD USA; Institute of Human Virology, University of Maryland School of Medicine, Baltimore, MD USA; Department of Public Health and Primary Care, University of Cambridge, Cambridge, UK

**Keywords:** Cervical Intraepithelial Neoplasia (CIN), Cervical cancer, Visual inspection, Acetic acid, Lugol’s Iodine, Recurrence, HIV, Thermo-coagulation, Cold coagulation, Ablation, Cervical Intraepithelial lesions, See-and-treat and Nigeria

## Abstract

**Background:**

The burden of cervical cancer remains huge globally, more so in sub-Saharan Africa. Effectiveness of screening, rates of recurrence following treatment and factors driving these in Africans have not been sufficiently studied. The purpose of this study therefore was to investigate factors associated with recurrence of cervical intraepithelial lesions following thermo-coagulation in HIV-positive and HIV-negative Nigerian women using Visual Inspection with Acetic Acid (VIA) or Lugol’s Iodine (VILI) for diagnosis.

**Methods:**

A retrospective cohort study was conducted, recruiting participants from the cervical cancer “see and treat” program of IHVN. Data from 6 sites collected over a 4-year period was used. Inclusion criteria were: age ≥18 years, baseline HIV status known, VIA or VILI positive and thermo-coagulation done. Logistic regression was performed to examine the proportion of women with recurrence and to examine factors associated with recurrence.

**Results:**

Out of 177 women included in study, 67.8 % (120/177) were HIV-positive and 32.2 % (57/177) were HIV-negative. Recurrence occurred in 16.4 % (29/177) of participants; this was 18.3 % (22/120) in HIV-positive women compared to 12.3 % (7/57) in HIV-negative women but this difference was not statistically significant (*p*-value 0.31). Women aged ≥30 years were much less likely to develop recurrence, adjusted OR = 0.34 (95 % CI = 0.13, 0.92). Among HIV-positive women, CD4 count <200cells/mm^3^ was associated with recurrence, adjusted OR = 5.47 (95 % CI = 1.24, 24.18).

**Conclusion:**

Recurrence of VIA or VILI positive lesions after thermo-coagulation occurs in a significant proportion of women. HIV-positive women with low CD4 counts are at increased risk of recurrent lesions and may be related to immunosuppression.

## Background

Cervical cancer is the fourth most common cancer in women and the fourth most common cause of cancer death worldwide [1]. While its incidence and mortality are falling in the developed world, mainly as a result of widespread adoption of cervical cancer screening covering about 75 % of at-risk women [2–4], in developing countries, where the burden is most severe, the incidence and mortality remain high and screening covers only 5 % of at-risk women [5, 6]. Cervical cancer is also one of the AIDS defining cancers and several studies suggest that its incidence is higher among HIV positive individuals [7–11]. In Sub-Saharan Africa the prevalence of HIV and cervical cancer are high. In Nigeria, the Age Standardized Incidence Rate (ASR) of cervical cancer was 34.5 per 100,000 in 2011, making it the second most common cancer in the country [12] and there were 3.2 million people living with HIV in 2013 (2nd highest globally) [13].

Cervical cancer occurs after a long asymptomatic premalignant phase, which is referred to as Cervical Intraepithelial Neoplasia (CIN). Majority of low-grade CIN lesions regress and only some high-grade CIN lesions progress to invasive cancer [14]. HIV infection is associated with higher risk of developing CIN; increased risk of developing a higher grade of disease and more rapid progression to malignant disease [15]. Treatment of high grade CIN using excisional or ablative methods are efficacious and substantially reduces risk of cervical cancer however they can be followed by recurrent disease [16].

Thermo-coagulation is an ablative treatment for premalignant lesions of the cervix developed by Kurt Semm in 1966 and is increasingly used worldwide. It uses electricity to generate temperatures of 100–120 °C for ablation of cervical lesions and can be used for all stages of CIN with minimal side effects. It is also safe, low-cost, has high client acceptance levels and is thus used in combination with visual inspection with acetic acid (VIA) or Lugol’s iodine (VILI) in low-resource settings as part of a “see and treat” strategy for cervical cancer screening [17]. Other methods of treating CIN include cryotherapy and excisional methods such as cold knife excision, loop excision of the transformation zone and laser conization.

Recurrence of CIN following treatment occurs in at least 15 % of cases even with the most effective excisional and ablative treatment modalities; and studies suggest that recurrence is associated with immunosuppression, HIV infection, positive endocervical margins, CIN grade, persistent HPV infection, glandular involvement and demographic factors [16, 18–26]. Excisional treatment of cervical lesions is more widely practiced in developed countries while ablative methods like thermo-coagulation and cryotherapy have gained ground in developing countries, yet recurrence of cervical lesions following treatment is a global problem, and studies have found ablative treatment to be of similar efficacy as excisional treatment irrespective of whether follow-up is based on histology or visual inspection [17]. Factors associated with recurrence after cervical ablative treatments may differ by populations and the specific method of ablation used [19, 24, 25, 27–29]. The risk of recurrence has been evaluated in developed countries but not so much in Africa which has a higher prevalence of HIV and cervical cancer. Previous African studies have focused on recurrence after treatment with techniques such as cryotherapy, electrocautery, laser cautery, LLETZ or cervical conization and there has been no previous study of recurrence after thermo-coagulation [30–32].

## Methods

We conducted an analysis of de-identified data of women who participated in the cervical cancer “see and treat” program of the Institute of Human Virology Nigeria (IHVN) that was implemented in collaboration with 6 hospitals—National Hospital Abuja (NHA), University of Abuja Teaching Hospital (UATH), Garki Hospital Abuja (GHA), Federal Medical Centre Keffi (FMCK), Aminu Kano Teaching Hospital (AKTH) and Mother and Child Hospital, Ondo (MCHO)—in different parts of Nigeria between 2010 and 2014. We evaluated early recurrence of VIA/VILI positive lesions in women who were previously screened and treated for cervical precancer using VIA/VILI.

Women older than 18 years were seen by trained nurses who obtained informed consent and collected demographic data, sexual behavior history, obstetrics and gynecological history, HIV status and if HIV-positive, time of HIV diagnosis and anti-retroviral treatment history. The nurses collected participants’ anthropometric measurements, performed a physical examination and a detailed pelvic examination. During the pelvic examination, they performed VIA and VILI for cervical intraepithelial lesions and those who tested positive were offered treatment by thermo-coagulation if their lesions met specific criteria: complete visualization of acetowhite lesion, acetowhite lesion occupying less than 3 quadrants or less than 75 % of the transformation zone, acetowhite lesion amenable to complete coverage by the tip of the cryoprobe, potential to achieve full contact between acetowhite lesion/transformation zone and the cervical probe; and no suspicion of cancer [33, 34]. Participants were followed up at 6-monthly intervals and monitored for recurrence. Women who did not return for their follow up appointments were contacted by telephone to schedule a visit for follow up examinations including evaluation for recurrence of VIA/VILI positive lesions.

Student’s *t*-test was used to compare continuous variables between HIV-positive and HIV-negative women while Fisher’s exact test was used for categorical variables. Logistic regression was performed to examine the proportion of women with recurrence and to examine factors associated with recurrence. Results are presented with 95 % confidence intervals. Analysis of the recurrence of cervical intraepithelial lesions was limited to women with at least 1 follow-up visit after thermo-coagulation. Multivariable logistic regression was used to identify risk factors for recurrence of cervical intraepithelial lesions. The following variables were evaluated for association with recurrent lesions - age, age at first intercourse, number of elective abortions, pregnancies and number of miscarriages, number of sexual partners, contraception use and CD4 count. Covariates with *p* value less than 0.10, along with biologically relevant variables, were included in the final multivariable model in which *p* value of less than 0.05 was considered statistically significant. Statistical analyses were performed using SAS® software (version 9.3).

## Results

**Fig. 1:**
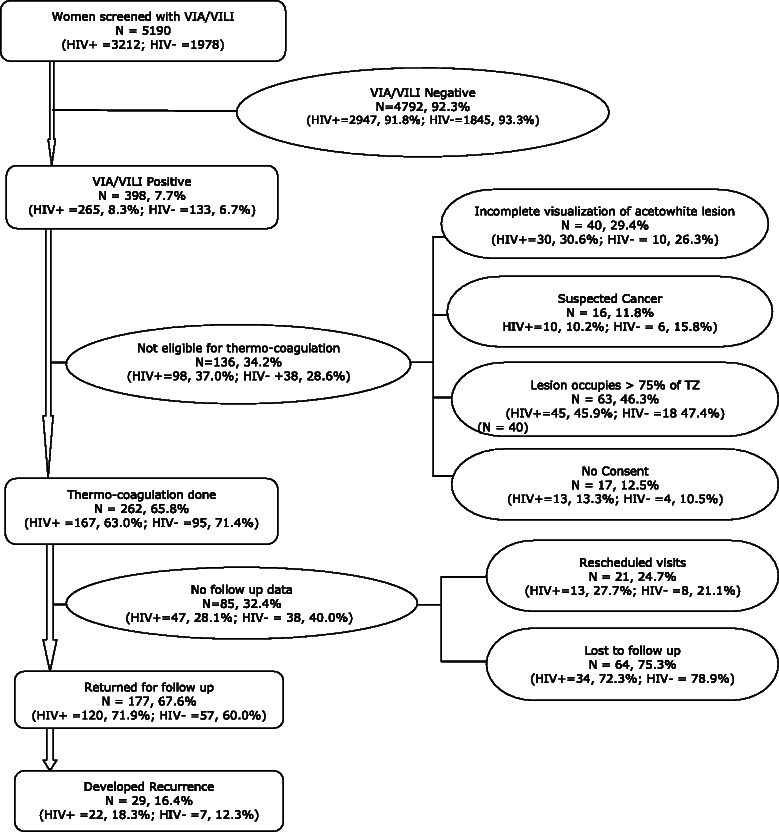
Flowchart of study participants

Out of a total of 5190 women screened by VIA or VILI over a 4-year period, the proportion of VIA/VILI positive lesions was 7.7 % (398/5190); this was 8.3 % (265/3212) for HIV-positive women and 6.7 % (133/1978) for HIV-negative women (*p*-value 0.05), see Table 1 and Fig. 1. Among women who were VIA/VILI positive, 65.8 % (262/398) had thermo-coagulation; of the remaining 136 (34.2 %), 109 (80.1 %) were ineligible for thermo-coagulation and subsequently referred to a gynecologist, 40 (29.4 %) had poorly visualized lesions, 16 (11.8 %) had suspected cancer, 63 (46.3 %) had lesions occupying more than three quarters of the transformation zone and 17 (12.5 %) did not consent. Note that the participants may have had more than one reason that made them ineligible for treatment by thermo-coagulation. Of those treated, 67.6 % (177/262) were followed up for at least 6 months (Fig. 1) and 32.4 % (85/262) were lost to follow-up. Fewer HIV-positive women, 28.1 % (47/167) compared to 40 % (38/95) of HIV-negative women (*p*-value 0.06) were lost to follow-up.

**Table 1:** Study sites by numbers screened and inclusion of participants

	SITE					
	NHA, *n* (%)	UATH, *n* (%)	MCHO, *n* (%)	GHA, *n* (%)	FMCK, *n* (%)	AKTH, *n* (%)
SCREENED, 5190	1979 (38.1)	1792 (34.5)	539 (10.4)	352 (6.8)	393 (7.6 %)	135 (2.6)
HIV Positive = 3212, *n* (%)	1563 (48.7)	1431 (44.6)	15 (0.4)	39 (1.2)	130 (4.0)	35 (1.1)
HIV Negative = 1978, *n* (%)	416 (21.0)	361 (18.3)	524 (26.5)	313 (15.8)	263 (13.3)	100 (5.1)
INCLUDED IN STUDY, 177	102 (57.6)	48 (27.1)	8 (4.5)	14 (8.0)	5 (2.8)	0 (0.0)
HIV Positive = 120, *n* (%)	79 (65.8)	38 (31.7)	1 (0.8)	2 (1.7)	0 (0.0)	0 (0.0)
HIV Negative = 57, *n* (%)	32 (40.3)	10 (18.5)	7 (12.3)	12 (21.1)	5 (8.8)	0 (0.0)
RECURRENCE, 29	13 (44.8)	13 (44.8)	1 (3.5)	2 (6.9)	0 (0.0)	0 (0.0)
HIV Positive = 22, *n* (%)	12 (54.5)	10 (45.5)	0 (0.0)	0 (0.0)	0 (0.0)	0 (0.0)
HIV Negative =7, *n* (%)	1 (14.3)	3 (42.8)	1 (14.3)	2 (28.6)	0 (0.0)	0 (0.0)

A total of 177 participants who had follow up visit at 6 months were included in the analysis of determinants of recurrent lesions after VIA/VILI and their baseline characteristics are shown in Table 2. The mean age (SD) of these participants was 34.9 (7.4) years and median follow up time was 531 days (IQR = 673). Most of these participants, 67.8 % (120/177), were HIV-positive and 32.2 % (57/177) were HIV-negative. Recurrence occurred in 16.4 % (29/177) and this was 18.3 % (22/120) in HIV-positive women compared to 12.3 % (7/57) in HIV-negative women but this difference was not statistically significant (*p*-value 0.31), see Table 3. Women aged 30 years or older were much less likely to develop recurrence, adjusted OR = 0.34 (95 % CI = 0.13, 0.92). Among HIV-positive women, CD4 cell count <200cells/mm3 was associated with recurrence, adjusted OR = 5.47 (95 % CI = 1.24, 24.18) (Table 4).

**Table 2:** Baseline characteristics by HIV status

Characteristics		HIV-positive (*N* = 120) *n* (%)	HIV-negative (*N* = 57) *n* (%)	*P* value*
Age				
< 30 years	*n* (%)	34.0 (28.3)	11.0 (19.3)	0.27
≥ 30 years	*n* (%)	86.0 (71.7)	46.0 (80.7)	
Age at first intercourse				
< 18 years	*n* (%)	36.0 (30.2)	10.0 (18.2)	0.10
≥ 18 years	*n* (%)	83.0 (69.8)	45.0 (81.8)	
Number of Sexual Partners				
≤ 1	*n* (%)	97.0 (82.2)	48.0 (85.7)	0.67
> 1	*n* (%)	21.0 (17.8)	8.0 (14.3)	
Elective Abortions				
Ever	*n* (%)	89.0 (76.7)	28.0 (56.0)	0.01*
Never	*n* (%)	27.0 (23.3)	22.0 (44.0)	
Number of Pregnancies				
< 5	*n* (%)	84.0 (70.6)	30.0 (53.6)	0.04*
≥ 5	*n* (%)	35.0 (29.4)	26.0 (46.4)	
Miscarriages				
Ever	*n* (%)	9.0 (8.3)	11.0 (21.6)	0.04*
Never	*n* (%)	99.0 (91.7)	40.0 (78.4)	
Body Mass Index (BMI), Mean (SD)		24.4 (5.2)	28.9 (6.5)	0.02*
Marital Status				
Ever Married	*n* (%)	88.0 (73.3)	47.0 (82.5)	0.26
Never Married	*n* (%)	32.0 (26.7)	10.0 (17.5)	
Highest Education				
Post-secondary	*n* (%)	52.0 (43.7)	36.0 (63.2)	0.02*
≤ Secondary	*n* (%)	67.0 (56.3)	21.0 (36.8)	
Contraceptive use				
Ever	*n* (%)	51.0 (42.5)	35.0 (61.4)	0.02*
Never	*n* (%)	69.0 (57.5)	22.0 (38.6)	
Genital Lesions (Warts, Sores or Ulcers)				
Yes	*n* (%)	9 (40.9)	5 (14.7)	0.06
No	*n* (%)	13 (59.1)	29 (85.3)	
Follow up duration in days, Median (Interquartile Range)^a^		793.5 (758.0)	371.0 (274.0)	<.0001*
Losses to follow-up	*n* (%)	47.0 (28.1)	38.0 (40.0)	0.06
CD4 Count (cells/mm^3^)				
< 200	*n* (%)	22.0 (23.7)	--	
≥ 200	*n* (%)	71.0 (76.3)		
HAART use				
Yes	*n* (%)	105.0 (87.5 %)	--	
No	*n* (%)	15.0 (12.5 %)		
HAART REGIMEN				
AZT-3TC-NVP	*n* (%)	49 (53.26)	--	
EFV-TDF-3TC	*n* (%)	18 (19.57)		
NVP-TDF-3TC	*n* (%)	7 (7.61)		
AZT-3TC-EFV	*n* (%)	4 (4.35)		
LPV/r-TDF-3TC	*n* (%)	4 (4.35)		
TDF-FTC-EFV	*n* (%)	4 (4.35)		
AZT-3TC-LPV/r	*n* (%)	2 (2.17)		
D4T-3TC-NVP	*n* (%)	2 (2.17)		
TDF-FTC-NVP	*n* (%)	2 (2.17)		

*AZT* Zidovudine, *3TC* Lamivudine, *NVP* Nevirapine, *EFV * Efavirenz, *TDF* Tenofovir, *LPV/r* Lopinavir/Ritonavir, *FTC* Emtricitabine, *D4T * Stavudine

*Statistically significant at 0.05 level

^a^Comparison of medians was done using Wilcoxon rank-sum test

**Table 3:** Univariate analysis of factors associated with recurrence (*N* = 177; recurrence, *n* = 29)

		Recurrence		Univariate	
				OR (95 % CI)	*P*-value
	*N*	*n*	(%)		
HIV status					
Negative	57	7	12.3	1.00	0.31
Positive	120	22	18.3	1.6 (0.60–4.00)	
Age category (years)					
< 30	45	14	31.1	1.00	0.003*
≥ 30	132	15	11.4	0.28 (0.12–0.65)	
Age at first intercourse					
< 18 years	46	9	19.6	1.00	0.46
≥ 18 years	128	19	14.9	0.71 (0.30–1.70)	
No of Sexual Partners					
≤ 1	145	24	16.6	1.00	0.93
> 1	29	5	17.3	1.05 (0.40–3.00)	
Elective Abortions:					
Never	49	5	10.2	1.00	0.12
Ever	117	24	20.5	2.27 (0.81–6.35)	
Number of Pregnancies					
< 5	114	22	19.3	1.00	0.19
≥ 5	61	7	11.5	0.54 (0.22–1.35)	
Miscarriages					
Never	139	27	19.4	1.00	0.15
Ever	20	1	5.0	0.22 (0.03–1.70)	
Marital Status					
Never Married	42	5	11.9	1.00	0.37
Ever Married	135	24	17.8	1.60 (0.57–4.50)	
Highest Education					
≤ Secondary	88	16	18.2	1.00	0.54
Post-secondary	88	13	14.8	0.78 (0.35–1.74)	
Contraceptive use					
Never	91	14	15.4	1.00	0.71
Ever	86	15	17.4	1.16 (0.53–2.58)	
Genital Lesions (Warts, Sores or Ulcers)					
No	42	7	16.7	1.00	0.14
Yes	14	5	35.7	2.78 (0.71–10.88)	
CD4 Count (cells/mm^3^)^a^					
≥ 200	71	8	11.3	1.00	0.03*
< 200	22	7	31.8	3.68 (1.15–11.73)	

^a^HIV positive*Statistically significant at 0.05 level

**Table 4:** Multivariable analysis of factors associated with recurrence (*N* = 177; recurrence *n* = 29)

	Multivariable	
	aOR (95 % CI)	*P*-value*
HIV status		
Negative	1.00	0.97
Positive	0.98 (0.30–3.17)	
Age category (years)		
< 30	1.00	0.03*
≥ 30	0.34 (0.13–0.92)	
CD4 Count (cells/mm^3^)		
≥ 200	1.00	0.04*
< 200	5.47 (1.24–24.18)	

*aOR* adjusted odds ratio

*Statistically significant at 0.05 level

## Discussion

In this study from Sub-Saharan Africa to evaluate efficacy of thermo-coagulation treatment for VIA/VILI positive cervical lesions, we found that one in six women had a recurrent lesion after treatment and no statistically significant difference existed between recurrence in HIV-positive women compared to HIV-negative women. We also found that women 30 years of age and older were much less likely to develop recurrence and among HIV-positive women, low CD4 count was significantly associated with risk of recurrence. Previous studies have found an association between HIV infection and increased risk of recurrence of CIN following excisional treatment [24, 25, 32].

Our finding that 18.3 % of HIV-positive women in our study had recurrent VIA/VILI positive lesions after treatment compares favorably with a study in Western Kenya that found recurrence in about 13 % of women who have had LLETZ within 12 months of follow up with colposcopy and biopsy [35]. Other studies have reported much higher recurrence in HIV-positive women following ablative or excisional treatment. Heard et al. [36] reported recurrence of 53.9 % after excisional treatment and follow-up with cytology, colposcopy and histology. Vuyst et al. [37] found recurrence (after follow-up by cytology) of High-grade Squamous Intraepithelial Lesions (HSIL) following cryotherapy to be 22.8 %. A systematic review by Tebeu et al. [26] reported recurrence of between 20 and 75 % after excisional treatment and having been followed up by histology; however, studies that utilized ablative treatments like cryotherapy and thermo-coagulation were excluded from analysis.

A few studies have compared recurrence between HIV-positive and HIV-negative women. Fruchter et al. [38] found recurrence to occur in 62.0 % of HIV-positive women compared to 18.0 % of HIV-negative women 36 months after treatment by either ablation or excision. Bambury et al. [39] reported a recurrence rate of 66.2 cases per 100 person-years in HIV-positive women compared to 3.0 per 100 person-years in HIV-negative women following treatment by thermo-coagulation or LLETZ. The main attraction of ablative or excisional treatment after VIA/VILI is the opportunity to reduce or eliminate repeated clinic visits. However, these recurrence rates suggest that these treatments may not be effective in the long run particularly in HIV positive women who are at increased risk of persistent HPV infection. The paradigmatic “see and treat” approach may therefore be inadequate and women with VIA/VILI positive lesions may require long term follow up and treatment of recurrent lesions.

We also found that women who are 30 years of age or older had lower likelihood of recurrence. Previous studies of HIV-positive women show a steady decline in prevalence of VIA/VILI positive lesions with increasing age, most noticeable after age 30 years [6]. This may be a reflection of reduced prevalence of HPV infection with increasing age. Host immunity may also affect the likelihood of recurrence [40], so HIV-positive women may be at higher risk of recurrence than HIV-negative women. We found that severe immunosuppression, as marked by CD4 cell count of <200/mm^3^ is associated with recurrence and this is consistent with the results of several studies of recurrence in HIV-positive women [35].

Our study provides some insight into the efficacy of thermo-coagulation treatment of VIA/VILI positive lesions in HIV-positive and HIV-negative African women and suggests a need for a review of screening policies on account of efficacy of treatment and risk of recurrence. HIV positive women with positive VIA/VILI positive lesions will require close long term follow up because of their higher risk of recurrence. The classical “see and treat” approach may be an insufficient screening strategy in this population because it may be associated with unacceptably high risk of recurrent lesions.

This study has limitations; it utilizes visual inspection (with Acetic acid and/or Lugol’s Iodine) without cytological or histological diagnosis. Although validity of VIA/VILI as method of screening for premalignant cervical lesions remains a subject of debate, a recent clinical trial by Huchko et al. [41] reveals that VIA/VILI provide acceptable results for cervical cancer screening in resource-poor settings. However, to improve validity of test results our study employed a Quality Assurance/Quality Control (QA/QC) mechanism. We utilized a web-based cervicography with gynecologist verification in addition to on-site specialist reviews and peer review among nurses as QA/QC methods, this process has previously been explained by Adebamowo et al [42]. Other limitations of this study include high rate of loss to follow up and short duration of follow up. It is possible that longer duration of follow up may reveal even higher recurrence in these women.

## Conclusion

Recurrence of VIA or VILI positive lesions after thermo-coagulation occurs in a significant proportion of women. HIV-positive women with low CD4 counts are at increased risk of recurrent lesions and may be related to immunosuppression.

More research on the efficacy of “see and treat” cervical cancer screening strategy is needed given the increasing adoption of VIA/VILI particularly as an adjunct to PEPFAR programs for HIV positive women.

### Ethics approval and consent to participate

The study was reviewed and approved by the National Health Research Committee (NHREC) of Nigeria (NHREC Protocol Number: NHREC/01/01/2007-19-09-2014, NHREC Approval Number: NHREC/01/01/2007-25/09/2014) and the Institutional Review Board (IRB) of the University of Maryland Baltimore (Reference number: HP-00061517).

### Consent for publication

Not applicable.

### Availability of data

The dataset(s) supporting the conclusions of this article are available upon request, please send requests to imanueloga@gmail.com.

## Abbreviations

CIN -: cervical intraepithelial neoplasia
HAART: highly active antiretroviral therapy
HIV: human immunodeficiency virus
VIA: visual inspection with acetic acid
VILI: visual inspection with Lugol’s iodine
